# Copper-Coated Polypropylene Filter Face Mask with SARS-CoV-2 Antiviral Ability

**DOI:** 10.3390/polym13091367

**Published:** 2021-04-22

**Authors:** Sunghoon Jung, Jun-Yeoung Yang, Eun-Yeon Byeon, Do-Geun Kim, Da-Gyum Lee, Sungweon Ryoo, Sanggu Lee, Cheol-Woong Shin, Ho Won Jang, Hyo Jung Kim, Seunghun Lee

**Affiliations:** 1 Department of Nano-Bio Convergence, Korea Institute of Materials Science, 797 Changwondae-ro, Changwon 51508, Korea; hypess@kims.re.kr (S.J.); yjy8184@kims.re.kr (J.-Y.Y.); whereks@kims.re.kr (E.-Y.B.); dogeunkim@kims.re.kr (D.-G.K.); 2 Department of Materials Science and Engineering, Seoul National University, Seoul 08826, Korea; 3 Department of Organic Material Science and Engineering, Pusan National University, Busan 609-755, Korea; hyojkim@pusan.ac.kr; 4 Clinical Research Centre, Masan National Tuberculosis Hospital, 215 Gaporo, Masanhappo-gu, Changwon 51755, Korea; viweon@korea.kr (D.-G.L.); viweon@naver.com (S.R.); 5 Building Energy Technology Center and Center for Climatic Environment Real-scale Testing, Korea Conformity Laboratories, 7 Jeongtong-ro Deoksan-myeon, Jincheon-gun, Chungcheongbuk-do 27872, Korea; oh239@kcl.re.kr (S.L.); scu1102@kcl.re.kr (C.-W.S.); 6 Department of Materials Science and Engineering, Research Institute of Advanced Materials, Seoul National University, Seoul 08826, Korea; hwjang@snu.ac.kr

**Keywords:** SARS-CoV-2, antiviral activity, copper film

## Abstract

Face masks will be used to prevent pandemic recurrence and outbreaks of mutant SARS-CoV-2 strains until mass immunity is confirmed. The polypropylene (PP) filter is a representative disposable mask material that traps virus-containing bioaerosols, preventing secondary transmission. In this study, a copper thin film (20 nm) was deposited via vacuum coating on a spunbond PP filter surrounding a KF94 face mask to provide additional protection and lower the risk of secondary transmission. Film adhesion was improved using oxygen ion beam pretreatment, resulting in cuprous oxide formation on the PP fiber without structural deformation. The copper-coated mask exhibited filtration efficiencies of 95.1 ± 1.32% and 91.6 ± 0.83% for NaCl and paraffin oil particles, respectively. SARS-CoV-2 inactivation was evaluated by transferring virus-containing media onto the copper-coated PP filters and subsequently adding Vero cells. Infection was verified using real-time polymerase chain reaction and immunochemical staining. Vero cells added after contact with the copper-coated mask did not express the RNA-dependent RNA polymerase and envelope genes of SARS-CoV-2. The SARS-CoV-2 nucleocapsid immunofluorescence results indicated a reduction in the amount of virus of more than 75%. Therefore, copper-coated antiviral PP filters could be key materials in personal protective equipment, as well as in air-conditioning systems.

## 1. Introduction

More than 2.6 million people have died because of the COVID-19 pandemic as of February 2021, and the spread of the virus continues to increase [1,2]. South Korea is currently facing a third outbreak of COVID-19 that began in mid-November 2020. The SARS-CoV-2 virus is transmitted via aerosols that are emitted as an infected person coughs or vocalizes, and the spread of viral infection due to aerosol outbreaks in indoor air has been widely reported [3,4,5,6,7]. Aerosols containing viruses often enter the human body via the respiratory tract (through the nose or mouth), and for this reason, wearing a face mask is an effective way to prevent SARS-CoV-2 infection [8,9,10]. The use of face masks has drastically increased worldwide since the beginning of the pandemic, which has led to a shortage of personal protective equipment (PPE) [11]. This shortage has threatened the safety of both medical staff and the general public, and it has led to medical staff in hospitals reusing masks that may have been contaminated with the virus during the treatment of suspected and confirmed COVID-19 patients [12]. Although mask supply problems have been resolved in some countries, others still face shortages [13]. The development of reusable PPE could resolve these issues while reducing the generation of micro-plastic waste caused by disposable masks [13,14,15,16].

Various studies have demonstrated that the risk of secondary transmission from the use of reusable masks can be minimized by the application of antiviral substances. For example, one type of mask has been found to be safe after as many as 50 uses when sterilized with high-temperature steam [17]. Washable fabrics such as silk have been proposed as a replacement for polymer-based N95 respirators [15,18]. Plasmonic photo-thermal heating has also shown promising results for the development of self-decontaminating respirators [19,20]. In addition, the antiviral effect of certain metals has been demonstrated using N95 respirators [21]. Specifically, the antiviral activity of copper is well-known [22,23,24,25]. Copper cations readily capture negatively-charged bacteria and viruses and can penetrate virus-infected bacteria. This activity prevents viral replication and infection functions. Silver and zinc oxide-based antimicrobial substances have also been widely used, and their application to respirators has been investigated [26].

Rapid global distribution of reusable antiviral masks will require mass production of masks that are demonstrated to be effective against SARS-CoV-2. The antiviral masks should be produced by a high-speed web process, the addition of the antiviral substances to the fiber surface should not degrade the filtration efficiency of the mask, and its antiviral effectiveness against the SARS-CoV-2 virus must be excellent. A solution that satisfies these three conditions is the application of a thin copper film coating to the mask filter by vacuum web coating. In the field of flexible electronics, copper thin films applied to the surfaces of polymer films exhibit excellent adhesion [27,28]. In addition, vacuum web-coating technology allows for high-speed and wide-width processing for mass production at low cost. Web-coating technology is a promising approach for the fabrication of antiviral copper-coated facial masks.

This study presents the development of copper-coated Korean filter 94 (KF94, equivalent to N95 respirators) masks that inactivate the SARS-CoV-2 virus upon contact with the mask surface. Among all the parts of the KF94 mask, the spunbond PP that covers it poses the greatest risk of exposing the wearer to the trapped virus. Therefore, a thin copper film was coated on the surface of the spunbond PP. The filtration efficiency and pressure drop of the copper-coated KF94 mask were evaluated, and its SARS-CoV-2 virus inactivation ability was demonstrated. 

## 2. Materials and Methods

### 2.1. Materials

Commercially available Kleenex KF94 masks (Kimberly-Clark) were used in this study.

### 2.2. Ion Beam Treatment and Copper Sputtering Deposition

Oxygen ion beams were generated using a closed-drift-type linear ion source (Home-built, Korea Institute of Materials Science (KIMS)) [29] that ionizes neutral oxygen gases based on electron drift via electromagnetic forces. The drift path was a linear closed loop with a length of 300 mm. Ion beam irradiation was conducted using a current of 100 mA, a voltage of 1.0 kV, and oxygen gas. 

Copper deposition was performed using a direct current (DC) magnetron sputtering system with high-purity copper (>99.9%) as the sputtering target. The working pressure during deposition was adjusted to ~1.0 mTorr using argon gas, and the power used for sputtering was 1000 W. The deposited copper film was ~20 nm thick.

### 2.3. Field-Emission Scanning Electron Microscopy

The surface shape of the spunbond PP fiber was observed using field-emission scanning electron microscopy (FE-SEM; JSM 6700F, JEOL, Tokyo, Japan) in secondary electron (SE) mode. The accelerating voltage was maintained at 5 kV.

### 2.4. Adhesion of Deposited Copper Thin Film

Adhesive tape (3M Scotch^®^ MagicTM tape 810, St. Paul, MN, USA) was used to evaluate the adhesion between the copper thin film and spunbond PP fiber in the copper-coated KF94 masks. Tape was applied to the mask surface, rubbed, and removed. Removal of the copper thin film with the tape was indicative of poor adhesion, while an intact copper-coated mask surface after tape removal was considered to demonstrate good adhesion. In order to quantify the amount of detached copper on tape surface after tape test, the tape surface was observed by scanning electron microscope (SEM; JSM6610LV, JEOL, Tokyo, Japan) with energy dispersive spectroscopy.

### 2.5. Filtration and Pressure Drop

The particle filtration efficiency and pressure drop of the coated mask were tested following the procedures described in the European standards EN143 and EN149. EN143 defines the classes of particle filter that can be attached to a facial mask, and EN149 defines the classes of filtering half masks or filtering face pieces. The standards test penetration of filter with dry NaCl and paraffin oil aerosols. Five uncoated KF94 masks and five copper-coated KF94 masks were tested using NaCl and paraffin oil particles.

### 2.6. Antiviral Effect against SARS-CoV-2 Virus

SARS-CoV-2 virus (National Culture Collection for Pathogens, NCCP 43328; 10^−5^ dilution, 500 μL) was transferred onto the uncoated and copper-coated spunbond PP surfaces for 1 h. The amount of virus was controlled prior to the contact reaction (Figure S3). The virus that had been in contact with the spunbond PP (200 μL) was transferred to Vero cells, and the samples were subsequently rocked every 15 min. The virus was removed and washed three times with phosphate-buffered saline (PBS). The medium of the Vero cells infected with the SARS-CoV-2 virus was exchanged once with fresh medium and the cells were cultured. 

### 2.7. Real-Time Polymerase Chain Reaction

Ribonucleic acid (RNA) was extracted from the Vero cells 48 h after they had been infected with the SARS-CoV-2 virus, using the QIAamp 96 Virus QIAcube HT Kit (Qiagen, Hilden, Germany). Real-time polymerase chain reaction (RT-PCR) was performed using a COVID-19 kit (STANDARD M n-CoV Real-Time Detection kit, SD Biosensor, Suwon, Korea). This kit targets the RdRp gene related to viral synthesis and the E gene related to the envelope. The threshold cycle (Ct) values of these genes were evaluated.

### 2.8. Immunochemical Staining

SARS-CoV-2 virus (NCCP 43326) was transferred onto the surface of the uncoated (*n* = 1) and copper-coated (*n* = 4) spunbond PP. The virus that had been in contact with the spunbond PP (200 μL) was transferred to Vero E6 cells and treated for 36 h (multiplicity of infection (MOI) = 0.01). Next, the sample was transferred to a plate and fixed with 4% paraformaldehyde for 1 h, before being treated with 1% Triton X-100 for 15 min for permeabilization of the cell membranes. The plate was washed with PBS, and then SARS-CoV nucleocapsid primary antibody (Rockland, 1:100) was added and the plate was maintained at 4 °C for 24 h. The secondary antibody Alexa Fluor^®^ 488 conjugate (Abcam, Cambridge, UK) was then added, and the plate was maintained at room temperature for 1 h, before the Hoechst 33342 stain (Thermo Fisher Scientific, Waltham, MA, USA) was applied at room temperature for 15 min. Fluorescent images were acquired using an automated microscope (LionHeart FX, Bio-Tek, Winooski, VT, USA). Image analysis was conducted to quantify the nucleocapsid expression using the proprietary Gene5 software.

### 2.9. X-ray Photoelectron Spectroscopy

The surface binding states of the copper thin films were analyzed using XPS (K-ALPHA+ XPS system, Thermo Fisher Scientific, Waltham, MA, USA) with a monochromated Al Kα source at 72 W and a spot size of 400 μm. Binding energies were computed using the energy of the C 1 s peak at 284.6 eV as a reference.

## 3. Results and Discussion

**Figure 1:**
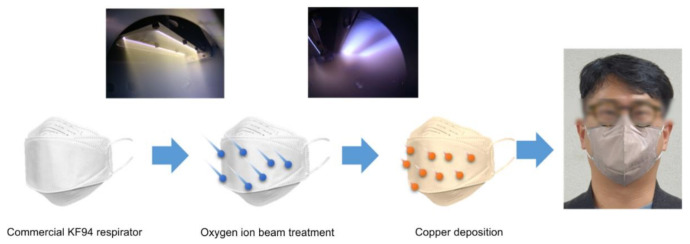
Copper-coated KF94 mask fabricated via vacuum surface treatments, including oxygen ion beam irradiation and copper sputtering deposition.

**Figure 2:**
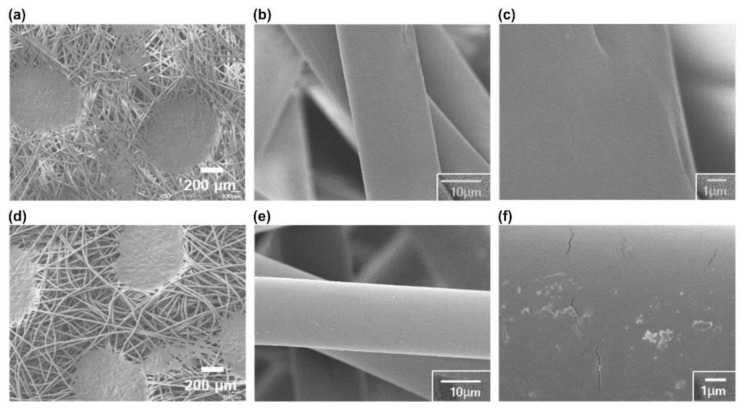
Field-emission scanning electron microscopy images of the spunbond polypropylene membrane of the KF94 mask (**a**–**c**) before and (**d**–**f**) after oxygen ion beam pretreatment and copper sputtering deposition.

The copper-coated KF94 masks were manufactured by irradiating the spunbond PP surface of commercially available KF94 masks with an oxygen ion beam at 600 ± 150 eV to give an irradiation dose of 2.0 × 10^16^ cm^−2^ (Figure 1). The polymer surface was prepared using oxygen ion beam irradiation to improve the adhesion of the metal thin films by creating a metal oxide interlayer [29]. The copper thin films (thickness, 20 nm) were deposited on the prepared spunbond PP via DC magnetron sputtering. To avoid thermal deformation, which would compromise the particle filtration efficiency, the spunbond PP fiber was not heated during treatment. The surface of the copper-coated spunbond PP was observed using FE-SEM in SE mode (Figure 2). The spunbond PP surface of the untreated KF94 mask consisted of PP non-woven fabric with a fiber diameter of 22.5 ± 1.5 μm. The fiber diameter was not changed after the ion-beam and sputtering processes. The distributions of fiber diameter are described in Figure S5. The copper-coated spunbond PP surface did not exhibit any structural deformation after ion beam irradiation and copper coating (Figure 2d–f). The cracks on Figure 2f is of copper thin films. These observations confirmed that the low energy associated with the oxygen ion beam irradiation (~0.1 J/cm^2^ s) did not cause thermal deformation. (Figure S6) A temperature-sensitive label demonstrated that the surface temperature increased to only 60 °C during irradiation. 

This damage-free surface treatment produced copper-coated masks with a similar pressure drop to an untreated KF94 mask. Specifically, the pressure drops of the copper-coated KF94 masks for NaCl (particle diameter 0.6 μm) and paraffin oil (particle diameter 0.4 μm) were 14.6 ± 1.2 and 14.0 ± 0.5 Pa, respectively, while those of the uncoated KF94 masks were 15.7 ± 0.6 and 14.5 ± 1.3 Pa, respectively (Table S1). The similarity in the pressure drop values indicates comparable particle filtration efficiency.

The good filtration efficiency of the copper-coated KF94 masks was confirmed via the NaCl and paraffin oil penetration test which referred to the EN143 and EN149 standards (Table 1) [30]. The filtration efficiencies of the copper-coated KF94 masks (*n* = 5) were 95.1 ± 1.32% and 91.6 ± 0.83% for NaCl and paraffin oil particles, respectively, and that of the uncoated KF94 masks (*n* = 5) was >99% for both particle types. Despite the respective 4.8% and 7.7% decrease for the NaCl and paraffin oil filtration efficiencies of the copper-coated KF94 respirators, this performance can still be considered good. These changes could be attributed to the ion beam irradiation and copper sputtering deposition processes. Charged particles from the plasma may have neutralized the KF94 masks, which has positive and negative electrostatic charges to capture fine particles by the Coulomb force.

**Figure 3:**
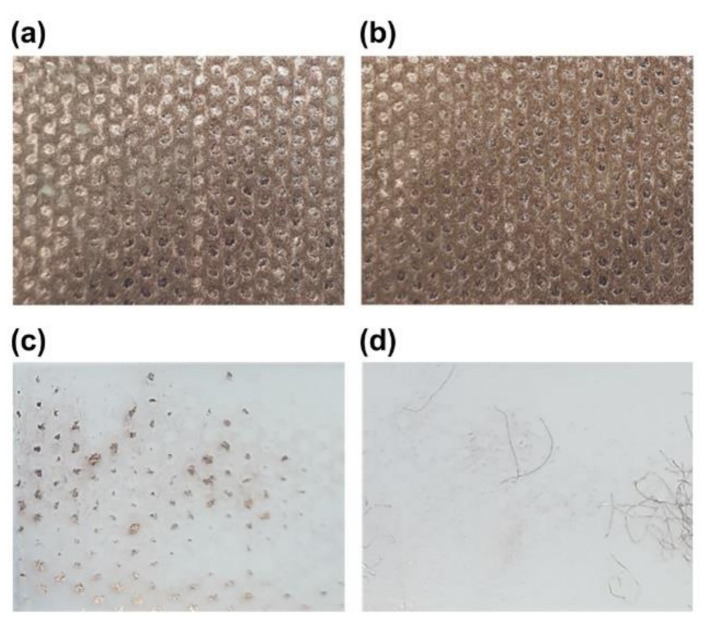
Tape adhesion testing of KF94 masks. Digital photographs of the copper-coated mask surfaces (**a**) without and (**b**) with oxygen ion beam pretreatment and of the surface of the removed tape (**c**) without and (**d**) with oxygen ion beam pretreatment.

Strong adhesion between the copper thin film and PP fibers ensures durability and user safety because detachment of copper nanoparticles from the mask surfaces may pose a toxicity threat. The adhesion of the copper thin film deposited on the spunbond PP surface was evaluated using a tape test (Figure 3). It was possible to detach the copper thin film using a tape adhesive from masks on which the oxygen ion beam treatment had not been conducted. However, the copper thin films exhibited excellent adhesion to the PP fiber when oxygen ion beam pretreatment was applied. Figure S4 shows SEM and elemental analysis images of tape surface after adhesion test. The copper films were detached from fiber in the non-treated sample. However, the fibers detached from the mask were observed in the ion beam treated mask, and copper was only observed on the fiber surface. This indicates that the adhesion of the copper thin film and PP fiber is improved by oxygen ion beam pretreatment.

The copper thin film was in the form of copper oxides due to its extremely thin nature (20 nm). Specifically, XPS analysis revealed that the surface of the copper-coated KF94 mask consisted of 75% cuprous oxide (Cu_2_O) and 25% cupric oxide (CuO) (Figure S1). Although oxygen gas was not added during copper sputtering deposition, copper oxide bonds were formed because of the oxygen bonds generated on the spunbond PP surface during the oxygen ion beam pretreatment. Pure copper thin films offer beneficial antiviral effects owing to the presence of copper ions. However, an antiviral coating consisting of a copper thin film with this thickness (~10–50 nm) can be bonded to the substrate more securely in the form of a copper oxide-type thin film. The antiviral effect of copper oxide was demonstrated in a previous study on a respiratory face mask with copper oxide nanoparticles, in which non-infectious human influenza A viral titers were recovered after 30 min [31]. However, this previously reported design posed a risk of detached copper nanoparticles being inhaled into the respiratory tract. In contrast, the deposition of a copper oxide thin film on the surface of the mask fiber is an optimal design for minimizing the detachment of toxic copper nanoparticles [32,33].

**Figure 4:**
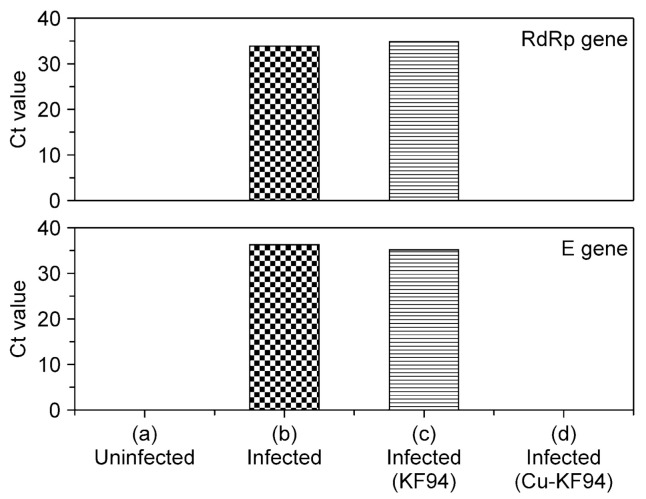
Threshold cycle (Ct) values from real-time polymerase chain reaction demonstrating that the copper-coated KF94 mask inactivated the SARS-CoV-2 virus. Plotted Ct values for RdRp and E genes in Vero cells (**a**) without SARS-CoV-2 virus infection, (**b**) with SARS-CoV-2 virus infection, (**c**) with SARS-CoV-2 virus infection after 1 h contact with uncoated K94 mask, and (**d**) with SARS-CoV-2 virus infection after 1 h of contact with copper-coated K94 mask.

The real-time PCR results for the RNA-dependent RNA polymerase (RdRp) and envelope (E) genes confirmed the antiviral activity of the copper-coated KF94 against the SARS-CoV-2 virus (Figure 4). The medium containing SARS-CoV-2 virus (NCCP 43328) was diluted by a factor of 10^−5^, and 500 μL was applied to the uncoated and copper-coated spunbond PP filters for 1 h (Figure S2). Vero cells were then infected using PP filters that had been treated with the virus for 1 h. A MOI of 0.01 was used for the infection media, as per maintenance media, i.e., only 2% fetal bovine serum (FBS) was added. Untreated spunbond PP and uninfected Vero cells were used as controls. The efficacies were evaluated based on quantification of viral copy numbers in the cell supernatant via quantitative real-time reverse transcription PCR (qRT-PCR). The findings were confirmed by visualization of virus nucleoprotein (NP) expression using immunofluorescence microscopy at 48 h post infection (p.i.). The cytopathic effect was not obvious at this point. Real-time analysis was performed on cell-associated viruses using probes for the RdRp and E genes. The threshold cycle (Ct) values of these SARS-CoV-2 genes after contact with the uncoated spunbond PP for 1 h were similar to those of control SARS-CoV-2 samples. However, the RdRp and E genes of SARS-CoV-2 were not detected after 1 h of contact with the copper-coated spunbond PP, as for the uninfected cells. Thus, qRT-PCR demonstrated that the copper-coated spunbond PP filters inactivated the SAR-CoV-2 virus.

**Figure 5:**
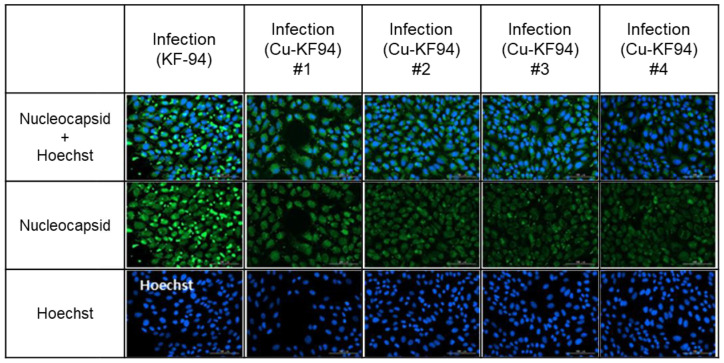
SARS-CoV-2 nucleocapsid expression after 1 h contact with uncoated (*n* = 1) and copper-coated (*n* = 4) KF94 masks (Cu-KF94).

The SARS-CoV-2 nucleocapsid immunochemical fluorescence signal was also used to demonstrate the antiviral ability of the copper-coated PP filters (Figure 5). Vero cells were infected with SARS-CoV-2, and immunofluorescent staining of the nucleocapsids was performed, with the nucleocapsid and Hoechst stains being observed as green and blue fluorescent emissions, respectively. The Hoechst stain was used to stain DNA, allowing identification of the cell nuclei. The uncoated KF94 control sample exhibited bright green nucleocapsids, while the nucleocapsid expression of SARS-CoV-2 was reduced by 75% in the copper-coated KF94 samples (*n* = 4). These in vitro results confirmed that the copper-coated PP filter had excellent antiviral activity against the SARS-CoV-2 virus. Accordingly, it is expected to be highly effective in the control of COVID-19 infection.

## 4. Conclusions

Copper-coated KF94 masks show promise as antiviral PPE for SARS-CoV-2 inactivation. In our study, their polymer fiber structure enabled the collection of bio-aerosol particles to trap the virus, while the antiviral copper thin film on the fiber surface reacted with the virus, resulting in its inactivation. Although detachment of the copper deposited on the polymer fiber surface would pose a hazard if it were to result in particles entering the respiratory tract, this was prevented by improved adhesion as a result of oxygen ion beam surface treatment, owing to the formation of copper–oxygen bonds between the copper film and the polymer surface. The power applied during this ion beam surface treatment and the copper thin film deposition was effectively controlled to prevent thermal damage to the polymer fiber. Thus, the filtration efficiency of the polymer fiber filter was maintained, and there was no significant difference between the pressure drop and particle filtration efficiencies of the copper-coated KF94 mask and untreated KF94 mask. However, the reduction of static electricity in the mask during treatment, owing to the fact that the ion beam and plasma surface treatment methods utilize electric charges, may partially reduce the filtration efficiency. The antiviral performance was evaluated by exposing Vero cells to the mask surface after 1 h of contact with the SARS-CoV-2 virus. Real-time PCR and immunostaining demonstrated the excellent antiviral performance of the copper-coated KF94 mask.

The moisture emitted in human breath, as well as the oxygen and moisture in the air, can promote oxidation of the deposited copper film. As copper oxide is known to inactivate the influenza A virus [31], oxidation of the copper thin film is not expected to compromise the antiviral performance of the material. However, the durability of the copper-deposited mask should be validated by analyzing the antiviral activity and degree of copper oxidation of the mask after extended exposure to high humidity. 

Copper deposition on spunbond PP can be performed using a vacuum web coater, which offers a fabrication speed of up to 100 m/min using multiple deposition sources. Future work we plan to undertake will explore the fabrication of lengths of up to 100 m of copper-coated spunbond PP textiles using a 300-mm-wide web coater. This continuous processing approach is expected to enable mass production of copper-coated KF94 masks.

## Figures and Tables

**Table 1:** NaCl and paraffin oil filtration performances of copper-coated and uncoated KF94 masks.

KF94 (Copper Coated)				KF94 (Reference)			
Sample No.	NaCl	Sample No.	Paraffin Oil	Sample No.	NaCl	Sample No.	Paraffin Oil
1	94.0	6	91.6	11	99.9	16	99.6
2	96.2	7	91.7	12	99.9	17	99.6
3	94.1	8	92.9	13	99.9	18	98.7
4	96.9	9	90.6	14	99.9	19	99.0
5	94.5	10	91.3	15	99.9	20	99.6
Average	95.1		91.6		99.9		99.3
Standard Deviation	1.3		0.8		0		0.4

## Data Availability

Not applicable.

## Supplementary Materials

The following are available online at https://www.mdpi.com/article/10.3390/polym13091367/s1, Figure S1: XPS analysis of the chemical binding status of copper thin films on KF94 masks. (a) peak fitting of Cu 2p3/2 peak, (b) details of the peak fitting, Figure S2: Digital photographs of the uncoated and copper-coated KF94 mask samples in contact with SARS-CoV-2 virus (10^−5^ dilution, 500 μL), Figure S3: Cycle threshold values for cell-associated virus used as an internal control, Figure S4: SEM and elemental analysis images of tape surface after adhesion test: (a) detached copper film on tape surface from mask sample without ion beam treatment (a–c), with ion beam treatment (d–f), Figure S5: Distributions of spunbond PP fiber diameter extracting 40 points from the 1 mm^2^ area: (a) before ion beam treatment, (b) after ion beam treatment and copper thin film deposition, Figure S6 Field-emission scanning electron microscopy images of the spunbond polypropylene membrane of the KF94 mask after oxygen ion beam treatment. (a) structure of spunbond PP filter, (b) PP fiber surface, (c) high-resolution image of PP fiber surface, Table S1: Pressure drop of KF94 masks measured using the EN143 and EN149 standard methods.
