# Supplementary material for: Copper-Coated Polypropylene Filter Face Mask with SARS-CoV-2 Antiviral Ability
Source: Polymers (Basel). 2021 Apr 22;13(9):1367. doi: 10.3390/polym13091367 (PMC8122733; doi:10.3390/polym13091367)
Supplement: Supplementary file 1 [file polymers-13-01367-s001.zip › polymers-1181655-supplementary.pdf]

SUPPLEMENTARY MATERIAL

Copper-Coated Polypropylene Filter Face Mask With SARS-CoV-2 Antiviral Ability

Sunghoon Jung [1,2], Jun-Yeoung Yang [1,6], Eun-Yeon Byeon [1], Do-geun Kim [1], Da-Gyum Lee [3], Sungweon Ryoo [3], Sanggu Lee [4], Cheol-Woong Shin [4], Ho Won Jang [5], Hyo Jung Kim [6] and Seunghun Lee [1]

[1] Department of Nano-Bio Convergence, Korea Institute of Materials Science, 797 Changwondae-ro, Changwon, 51508, South Korea
[2] Department of Materials Science and Engineering, Seoul National University, Seoul, 08826, South Korea
[3] Clinical Research Centre, Masan National Tuberculosis Hospital, 215 Gaporo, Masanhappo-gu, Changwon, 51755, South Korea
[4] Building Energy Technology Center and Center for Climatic Environment Real-scale Testing, Korea Conformity Laboratories, 7 Jeongtong-ro Deoksan-myeon, Jincheon-gun, Chungcheongbuk-do, 27872, South Korea
[5] Department of Materials Science and Engineering, Research Institute of Advanced Materials, Seoul National University, Seoul, 08826, South Korea
[6] Department of Organic Material Science and Engineering, Pusan National University, Busan, 609-755, South Korea
* Correspondence: seunghun@kims.re.kr; Tel.: (+82)-55-280-3512

(a)

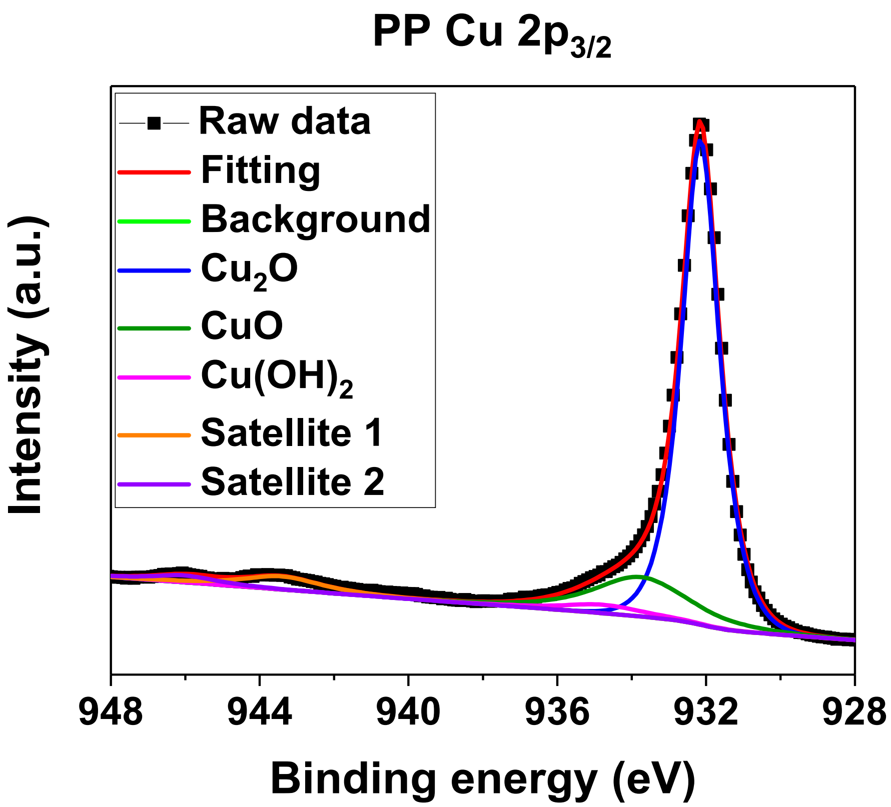

(b)

PP Cu 2p 3/2					
	B.E.	FWHM	%GL	Area	Area(%)
Cu_2O	932.15	1.2	80	709010.9	75.76
CuO	933.76	3	80	144429	15.43
$Cu(OH)_2$	934.75	2.5	80	25805.28	2.76
Satellite B1	943.4	2.5	80	40027.53	4.28
Satellite B2	946	2	80	16626.83	1.78
%Cu(I)	75.76				
%Cu(II)	24.24				

Figure S1. XPS analysis of the chemical binding status of copper thin films on KF94 masks. (**a**) peak fitting of Cu 2p3/2 peak, (**b**) details of the peak fitting.

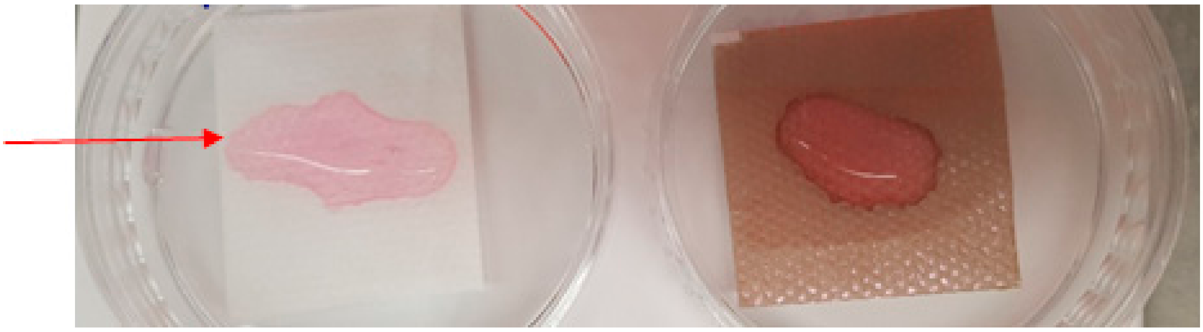

Figure S2. Digital photographs of the uncoated (left) and Cu-coated (right) KF94 mask samples in contact with SARS-CoV-2 virus (10-5 500 μL).

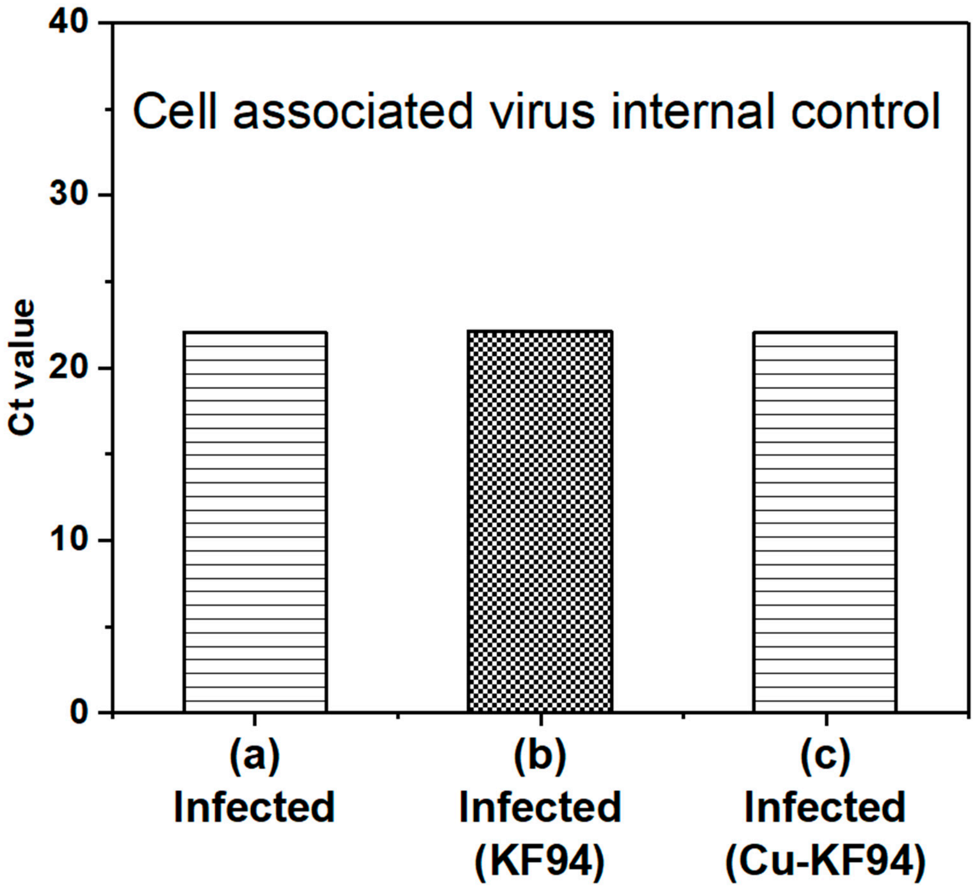

Figure S3. Cycle threshold values for cell associated virus used as an internal control.

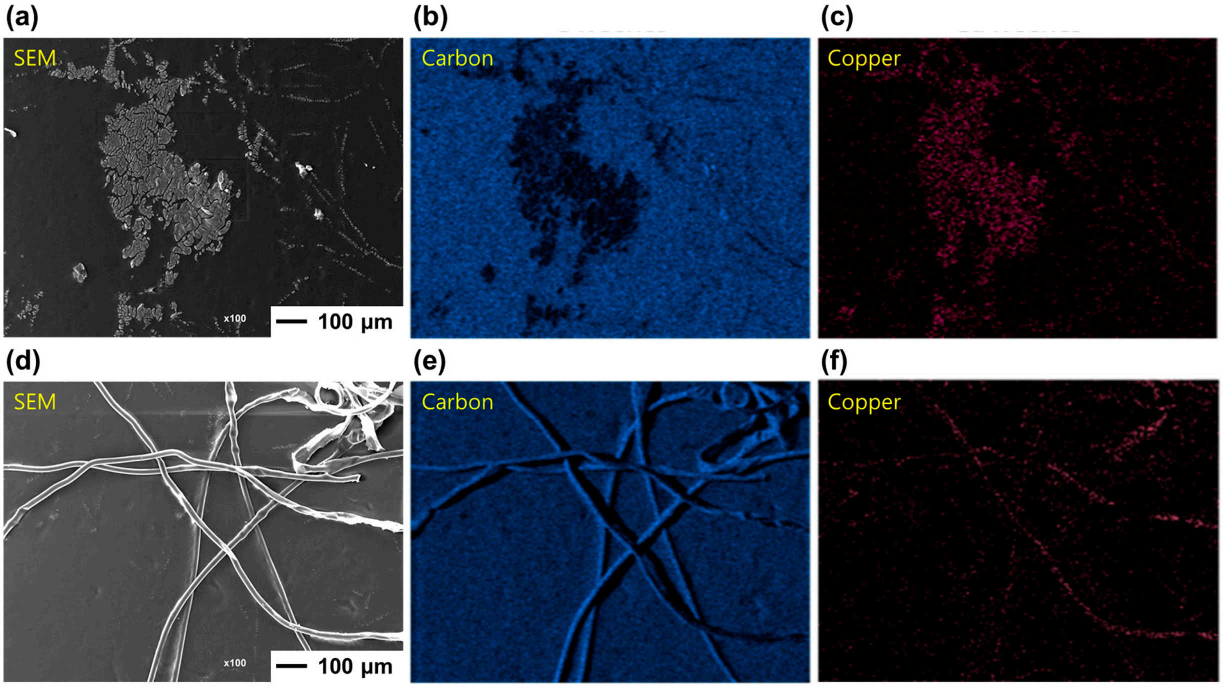

Figure S4. SEM and elemental analysis images of tape surface after adhesion test: (**a**) detached copper film on tape surface from mask sample without ion beam treatment (**a**–**c**), with ion beam treatment (**d**–**f**).

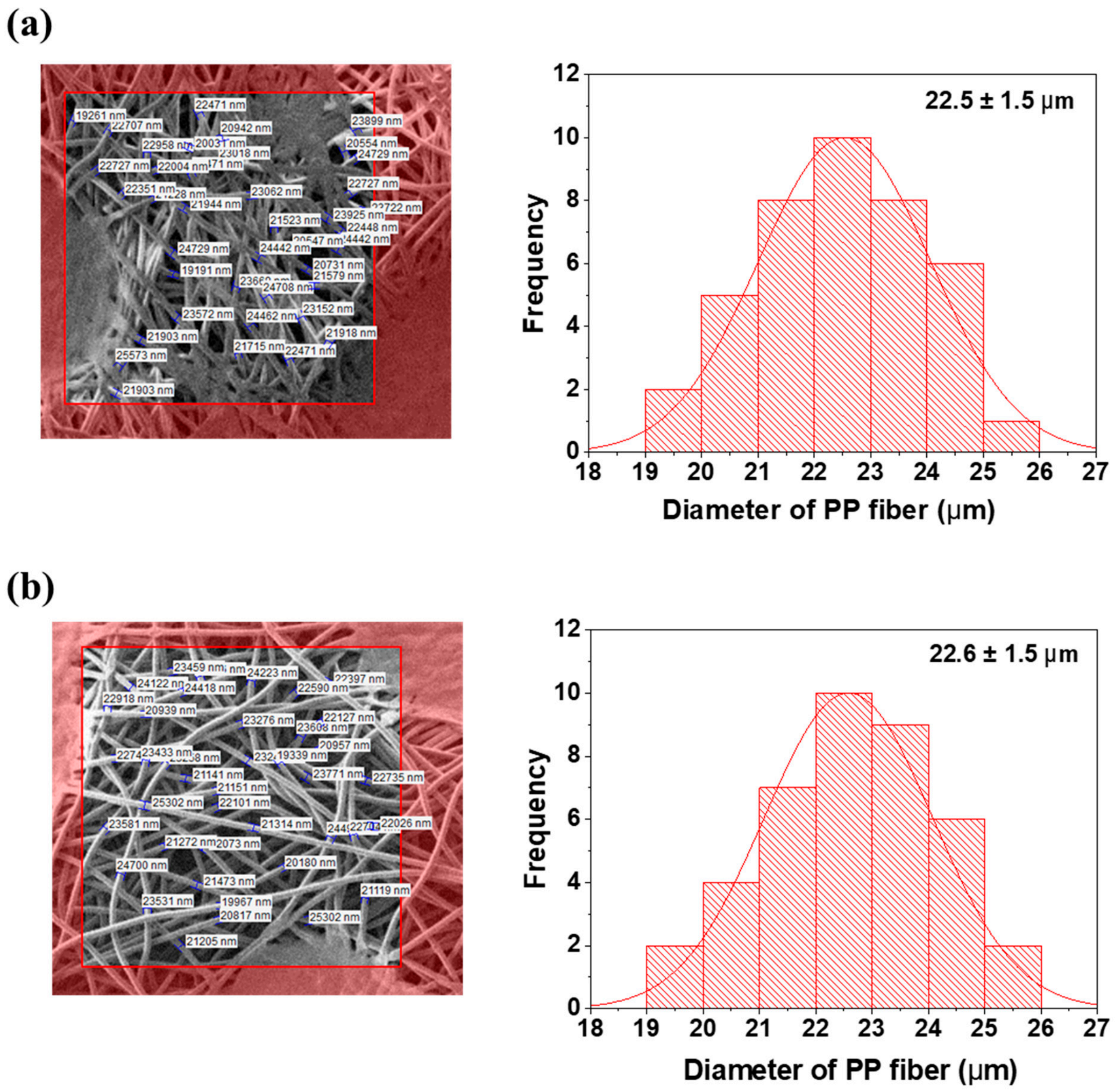

Figure S5. Distributions of spunbond PP fiber diameter extracting 40 points from the 1 mm² area: (**a**) before ion beam treatment, (**b**) after ion beam treatment and copper thin film deposition.

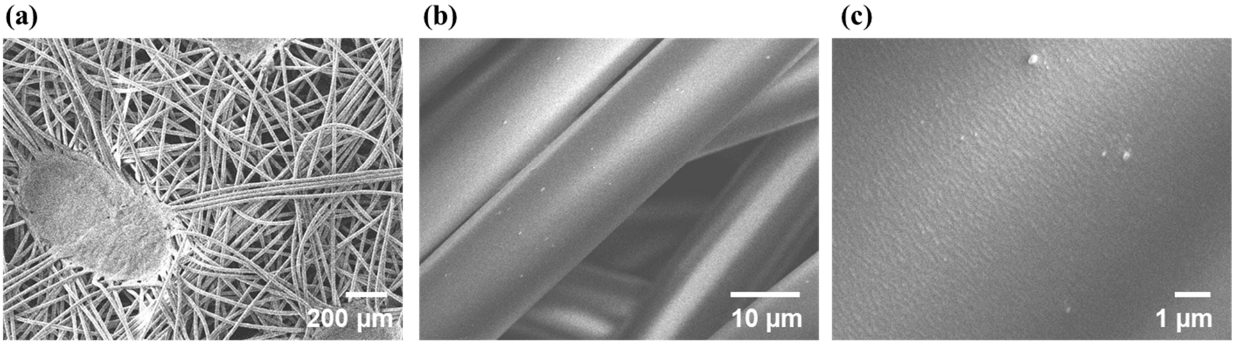

Figure S6. Field-emission scanning electron microscopy images of the spunbond polypropylene membrane of the KF94 mask after oxygen ion beam treatment. (a) structure of spunbond PP filter, (b) PP fiber surface, (c) high-resolution image of PP fiber surface.

Table S1. Pressure drop of KF94 masks using the EN143 and EN149 standard methods.

KF94 (Copper Coated)				KF94 (Reference)			
**Sample**	**NaCl**	**Sample**	**Paraffin Oil**	**Sample**	**NaCl**	**Sample**	**Paraffin Oil**
1	15.3	6	14.2	11	15	16	16.3
2	13.7	7	13.6	12	15.4	17	15.1
3	14.8	8	14.7	13	16.1	18	14.5
4	13.3	9	13.5	14	17.4	19	13.0
5	16.2	10	14.2	15	17.4	20	13.7
**Average**	14.6		14.0		15.7		14.5
**STDEV**	1.2		0.5		0.6		1.3
